# Theory of mind skills and peer relationships in children’s adjustment to preschool

**DOI:** 10.3389/fpsyg.2024.1373898

**Published:** 2024-07-24

**Authors:** Esra Ünlüer

**Affiliations:** Department of Preschool Education, Faculty of Education, Kocaeli University, Kocaeli, Türkiye

**Keywords:** theory of mind, peer relationship, school adjustment, school liking, preschool children

## Abstract

School adjustment affects children’s future lives in many ways. This study examined the relationship between ToM skills, peer relationships, and school adjustment. Specifically, this study determined whether preschool children’s school adjustment could be significantly predicted by theory of mind (ToM) skills and peer relationships. A total of 164 children aged 4 (34.5%), and 5 (38%) years of preschool attendance participated in the study. According to the research, children’s age, theory of mind, peer relations, and school adjustment are closely related. It was also found that the theory of mind significantly predicted school adjustment (school liking/avoidance) and that prosocial and aggressive behavior predicted school liking.

## Introduction

In the preschool period, which is an important stage in children’s development, children begin the process of adapting to school. At this stage, children learn to adapt to the skills, rules, and social norms necessary to establish relationships with their teachers and peers, share play materials, and follow school routines. Preschool is a developmentally appropriate period for learning the social skills that children will need for social adaptation in later years (Johnson et al., 2000). Children’s positive experiences in preschool environments can form a foundation that will enable them to establish social relationships with others; however, if they fail to overcome the difficulties experienced in this period, this can be seen as the cause of their adaptation problems later in life (Ladd et al., 1999).

### Development of theory of mind in early childhood

Theory of mind (ToM) is based on the child’s ability to predict and understand that the behavior of others is meaningful and intentional (Rowe et al., 2001). Children’s acquisition of theory of mind is a developmental process that enables them to have an increasingly sophisticated understanding of mental states (Wellman et al., 2001; Wellman and Liu, 2004). The process of acquiring the skills of the Theory of Mind begins in infancy (Repacholi and Gopnik, 1997). With the development of language skills in the second year of life, babies begin to talk about, interpret, and make sense of their own and others’ emotions and experiences (Grazzani-Gavazzi, 2004). According to Wellman and Liu (2004), the theory of mind development begins with developing an understanding of desires and then developing an understanding of beliefs. At this point, understanding belief is divided into two parts: understanding different beliefs and understanding false beliefs. From the age of three, children begin to understand that others may have different desires and that two people with different desires may feel different emotions in the same situation (Denham and Couchoud, 1990). Around the age of 4 years, the understanding of false beliefs begins to develop, and this development continues throughout childhood (Dore et al., 2018). Although rankings of theory of mind abilities that develop with age are universal, there are individual and cultural differences (Ertugrul Yasar, 2016).

There are two basic perspectives that attempt to explain the development of the theory of mind. One is the nativist perspective, which suggests that the theory of mind is largely innate. Another is the constructivist perspective (Westra and Carruthers, 2018). According to this perspective, children build their concepts of mind with the knowledge they gain about the minds of others, and as they acquire more and more data that their current concepts cannot explain, they may revise their concepts accordingly (Gopnik and Wellman, 2012, Wellman, 2014; Lane and Bowman, 2021). In line with this perspective, there are strong relationships between the knowledge children acquire from social experiences and their theory of mind (Lane and Bowman, 2021). The constructivist view is supported by evidence that the development of the theory of mind is influenced by social experience and language (Westra and Carruthers, 2018).

Recent functional imaging studies have strongly demonstrated that Theory of Mind (ToM) functions are facilitated by a highly organized and well-localized system in the brain (Drubach, 2008). Frith and Frith (2003) noted that social cognition processes are concentrated in specific regions of the brain, and structural or functional impairments in these regions can lead to significant deficits in ToM abilities. Brothers (1990) proposed a model for social cognition (encompassing ToM abilities) that consists of a three-node circuit connecting the orbitofrontal cortex, superior temporal sulcus, and amygdala. According to Brothers, if this circuit is disrupted at any point, it can result in autism, a disorder associated with significant deficits in ToM abilities.

### Peer relationships in early childhood

The concept of peer relationships involves those at a similar level of development, maturity, or age; it is expressed as a set of ongoing and reciprocal relationships between individuals with compatible values, lifestyles, social relationships, and backgrounds (Gulay, 2010). Children’s relationships with their peers change with age, with an increase in the amount of time spent with peers and the influence of play (Gulay, 2008). Peer interactions increase around the age of three, and as peer relationships increase around the age of four, children begin to experience rejection or acceptance (Oral, 2015). New communication skills acquired through peer interaction ensure the continuation of relationships and bring harmony and closeness. With their improved communication skills, they also produce behavioural solutions to the problems they experience with their peers (Hay, 2006). These relationships also impact social acceptance and competence in the years to be come (Walker, 2004).

Factors that influence competence in peer relationships have been studied for many years. The roots of individual differences in peer relationships lie in many variables such as family, especially parent-child relationships, and cognitive, social-emotional and social-cognitive development (Hazen and Brownell, 1999). Studies conducted with peer relationships support this. Studies have revealed the positive effects of parent-child relationship (McHale et al., 1999), cognitive development (Meece and Mize, 2009; Gulay et al., 2013), and social-emotional development (Gulay and Onder, 2013; Sen and Ozbey, 2017) on peer relationships.

### Association between theory of mind and peer relation

The theory of mind, one of the social cognitive abilities that affect peer relationships, assumes that individuals are aware of both their own cognitive state and the cognitive states of other people. The theory, which focuses on mental processes in the early years of life, is also extremely important for social development because of the impact of mental processes on social relationships (Gulay et al., 2013). Many studies conducted in recent years have focused on the relationship between children’s peer relationships and the theory of mind. These studies show that the theory of mind is positively related to prosocial behavior and increases peer acceptance (Caputi et al., 2012), whereas it is negatively related to negative behavior and leads to peer rejection (Banerjee and Watling, 2005; Suway et al., 2012).

Lane and Bowman (2021) suggest that when the role of social context in the development of theory of mind is taken into account, children who have more social interaction will develop their theory of mind skills more quickly because they have the opportunity to better understand the internal states of others. Conversely, less social interaction will lead to delayed theory of mind.

### Theory of mind and peer relationships in school adjustment

School adjustment is highly influential on children’s future lives in many ways. School adjustment predicts a range of positive outcomes for children, including future academic success and healthy socioemotional adjustment (Entwisle and Hayduk, 1988; Ramey et al., 1998; Ladd et al., 2000; Valeski and Stipek, 2001; Herndon et al., 2013; Blair and Raver, 2015). Considering the prevalence of problems related to school adaptation and their costs for the individual and society, cognitive and social competence are important interactional processes that should be examined in school adaptation studies. In particular, it is of interest for both developmental theory and educational policy to determine the factors that predict early school adjustment and children’s adaptation to new school environments (e.g., primary school entry) (Ladd, 2017).

Relationships with teachers, parents, and classmates have important effects on children’s ability to cope with many difficulties brought about by school adaptation, successfully adapt to this new environment, and like school (Ladd and Price, 1987; Baker, 2006; Ladd, 2017; Liew et al., 2019; Cheung et al., 2022). Children’s relationships with their peers in the classroom can be seen as an important support in the difficulties young children face in adapting to school (Coie et al., 1990; Wentzel, 1999; Betts et al., 2012; Ladd, 2017). Peers make up a large part of students’ social lives in the classroom and school (León and Liew, 2017), and forming close bonds with classmates can provide kindergarten students with the sense of security they need to explore and cope with the new environment (Johnson et al., 2000). There is strong evidence that children who establish high-quality relationships with their peers and are socially competent will actively participate in classroom activities, be academically successful, and adapt to school (Ladd et al., 1999, 2006; Caprara et al., 2000; Hay et al., 2004; Walker, 2004; Prinstein et al., 2005; Wood, 2007; Valiente et al., 2008; Nakamoto and Schwartz, 2010; Eggum-Wilkens et al., 2014; Hernández et al., 2016; Garner et al., 2020). Children who can establish good relationships with their peers have higher readiness for primary school (Polat and Atis-Akyol, 2016). Conversely, socially withdrawn children are often the target of peer rejection in the preschool period, which continues in primary school (Rubin et al., 1989). In addition, children who are not accepted do not participate in classroom activities, avoid school, and experience difficulties in academic areas (Buhs and Ladd, 2001; Wentzel, 2005; Klima and Repetti, 2008).

Early school adjustment involves a collection of academic, social, and regulatory skills (Pianta et al., 1997). Theory of mind, the ability to reason about the internal states of others, is an important and complex skill that influences skills in many areas of school adjustment (Brock et al., 2018). The theory of mind has significant relationships with academic (Blair and Razza, 2007; Astington and Pelletier, 2013), social (Watson et al., 1999; Capage and Watson, 2001), and regulatory skills (Aydin and Karakelle, 2016; Risnawati et al., 2023). The significant relationship between the Theory of Mind and the skills required for school adjustment may provide evidence that the Theory of Mind predicts school adjustment.

### The present study

As noted above, many studies have examined the theory of mind, peer relations, and school adjustment and their relationships with each other. To the best of our knowledge, no study has simultaneously addressed and clearly defined the role of children’s theory of mind and peer relations on school adjustment. The current study will contribute to our understanding of how young children’s ability to understand their internal mental states and their relationships with peers affect their adjustment to school. The ages of the children in our sample (4–5 years old), peer relations, and theory of mind guide our decision to test a model in which performance is expected to have a direct impact on school adjustment. First, consistent with the existing literature, we examined associations between children’s age, theory of mind, peer relationships, and school adjustment, even after controlling for common demographic characteristics. The age variable was included in the model to be evaluated. The second aim was to examine how the theory of mind and peer relations would affect school adjustment. We predicted that both theory of mind and peer relations would have direct effects on school adjustment.

## Method

### Participants

Participants in this study were selected using criterion sampling, a purposive sampling technique. Eligibility criteria included the absence of a) developmental delay, b) autism spectrum disorder, and c) sensory impairment. Developmental assessments were carried out in September by school counsellors using the Ministry of Education’s developmental assessment form. Accordingly, the study included 164 typically developing children aged 4 and 5 years. The children participating in the study were 78 (47.5%) 4 and 86 (52.4%) 5 years old. The children who participated in the study were 85 girls and 79 boys. In the survey conducted during the first four months of the 2023–2024 school year, children aged 4-5 have been attending school for at least two months. These children attend three different public kindergartens with similar socioeconomic levels. A total of 22 preschool teachers from these three different schools participated: 10 from the 4-year-old class, and 12 from the 5-year-old class. The teachers participating in the study had at least five years of teaching experience. While 36 (46.1%) of the 4-year-old children and 76 (88.3%) of the 5-year-old children had at least 1 year of school experience.

### Procedure

The start of the study was the approval of the research procedure by the Social Sciences and Humanities Ethics Committee of Kocaeli University. State kindergartens in the central district of Kocaeli were contacted to explain the aims of the study, the process, and the criteria required for the children participating in the study. The children who would participate in the study were identified through interviews with school principals and teachers. On the basis of these interviews, children were selected who had been approved by their teachers and who did not have a diagnosis (developmental delay, autism, etc.) in their individual files. The families and teachers of the children were given a participation form explaining the purpose and process of the study, and it was stated that a report about the study would be given at the end of the study.

The researcher performed the measurements in October, November, and December. The school visits started with meeting the 4–5 years old children in the last week of October, and the measurements continued throughout October, November. Completed by the teacher at the end of December. First, the School Liking and Avoidance Questionnaire was administered, starting with five sample items of the scale, and children who could not answer these items were not included in the study. Second, the Theory of Mind Battery was presented. Measurements, which took approximately 25 min, were performed individually in the guidance service’s room, and the children who volunteered to participate in the study were informed about the procedure again and reminded that they could terminate the study at any time. The Profile of Peer Relations (PPR) questionnaire was introduced to the classroom teacher and filled in by the teacher for each child.

### Measures

The School Liking and Avoidance Questionnaire, Theory of Mind Battery, and Profile of Peer Relations (PPR) Questionnaire were used as data collection tools.

### School liking and avoidance questionnaire

The scales developed by Ladd and Price (1987) and Ladd (2017) to determine children’s attitudes toward school were adapted for Turkish children by Nur and Arnas (2019). The SLAQ, which has 14 items, has a two-factor structure and evaluates school adjustment based on children’s perceptions. There are five sample items at the beginning of the questionnaire; these questions are not scored. The sub-dimension of school liking (items 1, 2, 4, 6, 7, 8, 10, 11, 12) includes children’s positive perceptions and feelings about school (items 2, 6, and 12 are calculated in reverse; Is school fun? Do you like being at school?) and the school avoidance sub-dimension (items 3, 5, 9, 13, 14) assesses children’s desire to avoid school (Do you want to stay at home instead of coming to school?). During individual interviews with the children, they were asked to rate the items on a three-point scale (“yes,” “sometimes,” or “no”), which were scored as 3, 2, and 1, respectively. Total score calculated by averaging the scores for each sub-dimension. High scores for the school liking subscale indicate positive feelings about school, whereas high scores for the avoidance subscale indicate a higher desire to avoid school. Cronbach’s alpha coefficient for the school liking sub-dimension of the SLAQ was determined to be.92 and.87. For the school avoidance sub-dimension (Nur and Arnas, 2019). In this study, two factors showed high internal consistency (Cronbach’s α = 0.88 and 0.81).

### Profile of peer relations (PPRs)

This inventory, developed by Walker et al. (2000), is used to assess each preschool child’s typical social and play behaviors with their peers (Walker et al., 2002; Walker, 2005). In the inventory, teachers were asked to rate children’s competence in identified social and play behaviors. The PPR, which has 25 items, included (a) items assessing the frequency of positive and negative play behaviors, such as cooperative play, verbal aggression, and physical aggression; (b) items detailing various strategies children may use when trying to participate in other children’s play; and (c) items related to the rate of engagement in conflict and conflict resolution strategies. Teachers were asked to rate the extent to which children exhibited certain behaviors on a 4-point Likert-type scale ranging from 1 (rarely) to 4 (almost always). Walker et al. (2000) identified three factors in the measure: aggressive or destructive behavior, prosocial behavior, and shy or withdrawn behavior. They reported that the three factors showed high internal consistency (Cronbach’s α = 0.91, 0.86, and 0.59). In this study, three factors showed high internal consistency (Cronbach’s α = 0.81, 0.79, and 0.61). The questionnaire is self-explanatory, dealing with behaviors that are easily visible within preschool environments. No prior training is required by qualified early childhood teachers to complete this inventory.

### Theory of mind tests

In this study, frequently used ToM tests with varying degrees of difficulty, as described by McAlister and Peterson (2006), were used. Three different types of ToM tests were used. These are false beliefs, appearance-reality, and pretend representation.

False Belief (FB): (a) the ‘Sally Ann’ task of Baron-Cohen et al. (1985) was used as a standardized invisible substitution task. This task was administered and scored faithfully to the original, except that the Turkish girls’ names Sare and Ayse were used instead of Sally and Ann. The trials began with the introduction of the dolls’ names and the materials to be used. Then, Sare put the marble in a closed basket and left the environment. In the first trial, the doll named Ayşe put the marble in a closed box, and in the second trial, she put it in the tester’s pocket. Sare returned asking for her marble. The child was asked the question “Where will Sare look for her marble first?” The answer “She will look for it in the closed box” for two trails is 1 point. She scored 1 point, with a maximum of 2. (b) A standardized deceptive container task was used, the test developed by Gopnik and Astington (1988). In this task, the child was shown a closed band-aid box and asked what was inside. Then, the child was shown the pencils inside the band-aid box, and the box was closed again. Regarding the belief of others, the child was asked, [classmate’s name] will come next. He/she does not know what is in this box. When I show him/her the box, what will he/she tell me about what is in the box? In relation to his/her own false belief, he/she was asked, “When I first showed this box to you—before you saw the contents of the box—what was in the box?’. For the two questions, the answer “bandaid” is worth 1 point. The maximum score for this task is 2. The sum of the scores obtained from these two tasks constitutes the total false belief (TFB) score. The total can vary between 0 and 4. Two false belief scores were found to be *r* = 0.50, *p* < 0.001 in the McAlister and Peterson (2006) study and *r* = 0.48, *p* < 0.001in this study.

Pretend Representation (PR): The child was shown a real potato. The child was asked to pretend that the potato was soap. After a short period of role play, the game was finished and the child was asked: “What is this really?’ (1 point for potato answer, 0 point for soap answer) and “What did we pretend it was?” (1 point for soap answer, 0 point for potato answer). After the child answered, a real soap and an irrelevant object (tennis ball) were placed on the table. “Which one did we pretend to be soap?” (1 point if it shows potato, 0 points if it shows soap or other objects) and “Which one is really soap?” (1 if he/she shows soap, 0 if he/she shows potato or other objects). The same test was repeated with a real banana. The banana was used as a phone in the role play, and a real cell phone was used at the end of the test. The total score was halved so that the children’s total score for each task was 0-4 points. The total score was halved so that the children’s scores for the two tasks would be similar to that of the false belief task. Pretend representation scores were found to be *r* = 0.31, *p* < 0.01 in the McAlister and Peterson (2006) study and *r* = 0.35, *p* < 0.01 in this study.

Appearance-Reality (AR): This test was based on Flavell (1986) procedure. In the first task, unlike the original, an orange was used instead of an apple. The children were shown an orange made of wax and asked what it was. After the children answered “orange,” a candle was lit and it was shown that it was actually a candle. They were then asked “What is this really?” (reality test) and “When you look at this now, does it look like a [candle] or [an orange]?” (appearance test). The child who answered both test questions correctly scored 1 point, but the child who gave only one correct answer scored 0 points. The same trial was repeated with a ballpoint pen that actually looked like a flower. The sum of the scores from these two tasks is the total AR score. The total score can range from 0 to 2. The two appearance-reality scores were found to be *r* = 0.34, *p* < 0.01 in the McAlister and Peterson (2006) study and *r* = 0.38, *p* < 0.001 in this study.

### Total ToM score

To calculate the total ToM score, first the relationships between the three tasks were examined and then the total score was calculated. The total FB scores of the children were found to be significantly correlated with AR (*r* = 0.69, *p* < 0.001) and PR (*r* = 0.56, *p* < 0.001); the total PR score was found to be significantly correlated with the total AR score (*r* = 0.23, *p* < 0.001). To create the total ToM score, first, the sums of FB and PR totals were divided by 4 and the AR total by 2 to compute the proportion score for each component. The total score was averaged to create a total ToM score that could vary between 0 and 1. In this study, Total ToM showed acceptable internal consistency (Cronbach’s α = 0.76).

### Statistical analyses

All statistical analyses were performed using SPSS version 22. Descriptive analyses were performed on the scores of the children using the data collection tools, and the mean and standard deviation were calculated. To test the normality of the distribution of the data, Skewness and Kurtosis Test was performed ToM (Skewness −0.089; Kurtosis −0.675), School Liking (Skewness −0.689; Kurtosis −0.961), School Avoidance (Skewness 0.717; − Kurtosis −0.482), Aggressive/Disruptive Behaviour (Skewness −0.183; Kurtosis −0.746), Prosocial Behaviour (Skewness.307; Kurtosis −1.274), Shy/Withdrawn Behaviour (Skewness 0.358; Kurtosis −0.860) and it was determined that the distribution was between 1.5 and 1.5. Because the data were within the normal distribution range, parametric tests were applied. The Pearson Product Moment Correlation Coefficient was calculated to reveal the relationship among children’s age, ToM performance, peer relations, and school adjustment. Multiple linear regression analysis was performed to determine the predictive power of ToM and peer relations on school adjustment.

## Results

### Descriptive analysis

The descriptive statistics of the study variables are presented in Table 1.

**TABLE 1:** Children’s performance on the ToM, peer relations, and school adjustment.

			Age Group		
			4 age	5 age	
		Range	Main Score (SD)		Total
Theory of Mind	False Belief	0–4	2.12 (0.93)	3 (0.92)	
	Pretend Represent.	1–4	3.3 (0.97)	3.46 (0.74)	
	Appearance reality	0–2	0.57 (0.71)	1.51 (0.5)	
	Total ToM	0–1	0.53 (0.17)	0.79 (0.16)	
Profile of Peer Relations	Aggressive/Disruptive	1–4	1.61 (0.41)	1.51 (0.31)	
	Prosocial Behavior	1–4	2.98 (0.33)	3.42 (0.46)	
	Shy/Withdrawn	1–4	1.77 (0.42)	1.40 (0.4)	
School Adjustment	School liking	9–27	22.05 (2.34)	26 (1.16)	
	School avoidance	5–15	5.9 (1.27)	9.4 (2.12)	

#### Correlational analyses

Table 2 provides an overview of the correlations among the variables included in the study.

**TABLE 2:** Correlations among children’s age, ToM, peer relations, and school adjustment.

	Theory of Mind	Aggressive /Disruptive	Prosocial Behavior	Shy /Withdrawn	School liking	School avoidance
Age	0.626**	−0.108	0.462**	−0.392**	0.747**	−0.728**
Theory of Mind		−0.266**	0.784**	−0.704**	0.717**	−0.671**
Aggressive/Disruptive			−0.294**	0.034	−0.387**	0.184*
Prosocial Behavior				−0.689**	0.666**	−0.459**
Shy/Withdrawn					−0.443**	0.463**
School liking						−0.820**
School avoidance						–

*p < 0.01 **p < 0.001.

Table 2 shows that children’s age is significantly positively related to ToM (*p* < 0.001), prosocial behavior (*p* < 0.001) and school liking (*p* < 0.001), and is significantly negatively related to shy or withdrawn behavior (p < 0.001) and school avoidance (*p* < 0.001).

#### Regression analysis

A regression analysis was conducted to investigate whether children’s peer relationships and Theory of Mind scores explain the variance in school liking and avoidance scores. The regression model was designed to evaluate the variance in school liking/avoidance scores in three successive steps. In the first step (Step 1), the role of age was assessed. Subsequently (Step 2), the impact of three dimensions of peer relationships (prosocial, aggressive, and shy behaviors) was examined by entering them into the regression model. Finally, in Step 3, the effect of Theory of Mind was evaluated.

The age of the children was entered in Step 1. The model was statistically significant, F(1,162) = 204.67, *p* < 0.000, explaining approximately 55% of the variance. Only the age variable accounted for the variability in school like scores (β = 0.74, *p* < 0.001). In Step 2, F(4,159) = 111.07, *p* < 0.000, the inclusion of three dimensions of peer relationships resulted in a statistically significant increase in explained variance (R_2_ = 0.73). Specifically, it was found that the dimensions of prosocial behaviors (β = 0.25, *p* < 0.000) and aggressive behaviors (β = −0.12, *p* < 0.000) of peer relationships had a significant impact on school liking scores. Finally, when Theory of Mind (ToM) was entered into the regression model, F(5,158) = 91.85, *p* < 0.000, it increased the explained variance by 1% (R_2_ = 0.74). The beta weight for ToM is (β = 0.23, *p* < 0.05) (see Table 3). This result indicates that prosocial and aggressive behaviors, along with Theory of Mind, predict school liking scores.

**TABLE 3:** Regression outcomes for target variable School Liking.

	β	SE	t	p
**Step 1**				
Age	0.747	0.280	14.307	0.000
**Step 2**				
Age	0.57	0.248	12.340	0.000
Aggressive/Disruptive	−0.219	0.350	−4.989	0.000
Prosocial Behavior	0.366	0.361	5.822	0.000
Shy/Withdrawn	−0.041	0.345	0.697	0.487
**Step 3**				
Age	0.516	0.279	9.911	0.000
Aggressive/Disruptive	−0.201	0.353	−4.546	0.000
Prosocial Behavior	0.296	0.402	4.224	0.000
Shy/Withdrawn	0.093	0.370	1.487	0.139
ToM	0.174	1.012	2.162	0.03

The age of the children was entered together in Step 1. The model was statistically significant, F(1,162) = 182.77, *p* < 0.000, explaining approximately 53% of the variance. Only the age variable accounted for the variability in school avoidance scores (β = −0.72, *p* < 0.000). In Step 2, F(4,2159) = 54.68, p < 0.000, the inclusion of three dimensions of peer relationships resulted in a statistically significant increase in explained variance (R_2_ = 0.57). Specifically, it was found that the dimension of peer relationships’ shyness (β = 0.22, *p* < 0.001) had a significant impact on school avoidance. Finally, when Theory of Mind (ToM) was entered into the regression model, F(5,220) = 51.64, *p* < 0.000, it increased the explained variance by 5% (R_2_ = 0.62). The beta weight for ToM (β = −0.40, *p* < 0.000) indicated that it should be considered a robust determinant of school avoidance scores (see Table 4). When accounting for the effect of Theory of Mind in the model, it can be observed that the relationship between shyness and school avoidance scores loses its statistical significance. This result demonstrates the significant role of Theory of Mind in the relationship between shyness and school avoidance.

**TABLE 4:** Regression outcomes for target variable School Avoidance.

	β	SE	t	p
**Step 1**				
Age	−0.728	0.264	−13.519	0.000
**Step 2**				
Age	−0.639	0.286	−10.934	0.000
Aggressive/Disruptive	0.114	0.405	2.063	0.061
Prosocial Behavior	0.025	0.416	0.310	0.757
Shy/Withdrawn	0.225	0.399	3.061	0.003
**Step 3**				
Age	−0.512	0.310	−8.078	0.000
Aggressive/Disruptive	0.072	0.393	1.342	0.181
Prosocial Behavior	0.188	0.447	2.205	0.079
Shy/Withdrawn	0.103	0.412	1.357	0.177
ToM	−0.406	1.126	−4.148	0.000

## Discussion

The main aim of this study was to investigate both theory of mind and peer relations in children’s adjustment to school in early childhood. Because of the study, two main findings were obtained. First, there were significant relationships between the investigated variables even after controlling for age. Second, it was found that theory of mind significantly predicted school adjustment (school liking/avoidance) regardless of age, prosocial behaviors positively and aggressive behaviors negatively predicted school liking.

### Relationships among age, theory of mind, peer relation, and school adjustment

In this study, statistically significant correlations were observed between the investigated variables. Consistent with the literature, this study shows that overall performance on ToM tasks increases with age (Gopnik and Slaughter, 1991; Wellman et al., 2001; Slaughter et al., 2002; Newton and Jenvey, 2011; Conte et al., 2019). The study found, as expected, that the Theory of Mind was strongly related to peer relationships. Consistent with previous research, there were positive relationships between the theory of mind and prosocial behavior and negative relationships between aggression and shyness. Imuta et al. (2016) reviewed 76 studies conducted with children aged 2–12 years and found a positive relationship between ToM and prosocial behaviors, similar to our study. Early understanding of the basic concepts of the theory of mind supports the development of prosocial behavior and indirectly mediates peer acceptance (Denham et al., 1990; Capage and Watson, 2001; Caputi et al., 2012). The basic concepts in the theory of mind are beliefs, desires, and intentions that are used to understand the behavior of others and predict how they will behave (Kloo et al., 2010). The theory of mind is necessary for children to learn basic concepts of prosocial behavior such as sharing and cooperation. Delays in the acquisition of these basic concepts are thought to underlie aggressive and destructive behavior in children (Capage and Watson, 2001; Wellman et al., 2011; Shakoor et al., 2012; Lane et al., 2013).

Previous studies on shy children have shown different results regarding their theory of mind abilities. In some studies, introverted children learn the contents of others’ minds through social observation and have a high theory of mind (Wellman et al., 2011; LaBounty et al., 2017), whereas in others, behavioral reactions, social anxiety, and fear-related behaviors are reported. Shy children have a less developed theory of mind (Banerjee and Henderson, 2001; Suway et al., 2012, Lane et al., 2013). Shy individuals, who are cautious and anxious about the situations created by social environments, experience a conflict of approach and avoidance in such situations. While shy children want to engage in social interaction, they avoid interaction because of the fear and anxiety they experience (Coplan et al., 2004; Coplan and Armer, 2007). Avoidance of interaction causes shy children to have many problems adjusting to school and to avoid school (Wu et al., 2015).

### ToM and peer relationships in school adjustment

In this study, theory of mind and prosocial behavior positively predicted school liking and aggressive behavior negatively; only theory of mind was found to negatively predict school avoidance. These findings agree with previous studies showing that theory of mind (Bolnick, 2008) and peer relationships (Gulay and Erten, 2013; Aydogdu, 2022) have important effects on children’s adjustment to school. Pre-school education presents many new challenges for children, such as meeting the demands of the environment, building relationships with teachers and peers, expanding their social circle, and dealing with social relationship problems. The theory of mind facilitates school adjustment by helping to develop some of the basic skills needed to cope with these difficulties (Chi et al., 2018; Brock et al., 2019). In addition, Dunn (1995) stated that children’s adjustment to school, which is a social environment, may be related to some characteristics of their understanding of others’ internal states, and that children with better ability to understand others should like school more. There are studies that show that the Theory of Mind has strong relationships with other skills that affect school adjustment, apart from social relationships. The strong relationships of the Theory of Mind with cognitive skills (Lockl and Schneider, 2007; Lecce et al., 2017), such as metacognitive and language skills (Conte et al., 2019), support its effect on school adjustment.

Positive peer relationships in the preschool years support children in the school environment and help them adjust to school. This study also shows that prosocial and aggressive behaviors are effective in children’s liking of school. This finding of our study is in line with the results of previous studies. In fact, Gulay and Erten (2013) and Aydogdu (2022) reported in their studies with preschool children that peer relationships predict liking for school. Children who display aggressive and disruptive behavior are often rejected by their peers (Slaughter et al., 2002; Gunnar et al., 2003) and have difficulty adjusting to school. In contrast, children who engage in positive social behavior are accepted by their peers and adapt to school more easily (Chung-Hall and Chen, 2010). In addition, this study showed that shyness did not have a direct effect on liking school. This finding was not consistent with studies showing that the combination of shyness and reserve predicts a range of social, emotional, and school adjustment difficulties in preschool (Chen et al., 1995; Coplan et al., 2008; Wu et al., 2015). Unlike previous studies examining the relationship between school adjustment and withdrawn behavior, this study includes the variable of theory of mind closely associated with withdrawn behaviors (Banerjee and Henderson, 2001). This suggests the possibility that shyness predicts school adjustment through the theory of mind.

### Implications and limitations

In summary, this study found that theory of mind, peer relations, and school adjustment are closely related; that liking school, which is a sub-dimension of school adjustment, is predicted by theory of mind as well as prosocial and aggressive behavior; and that the most important predictor of young children’s school adjustment is theory of mind. To the best of our knowledge, this is the first study to examine the relationships between these variables in a single model with children under the age of six. It is recognized that this study has some strengths and some limitations.

First, the cross-sectional nature of the study is a limitation in understanding the changes and effects of the variables in subsequent years. Second, the fact that the data in the study were collected very soon after the children started school and the possibility that the teachers, who are the source of information about the children’s peer relationships, do not know the children well enough is another important limitation of the study. Although this study contributes to our understanding of young children’s theory of mind, peer relationships, school adjustment, and their interrelationships, a longitudinal study in which measurements are repeated at different times will contribute to our understanding of long-term changes or continuity in these variables. This study attempted to determine whether there is a direct relationship between the theory of mind, peer relationships, and school adjustment. However, future studies should examine a model that incorporates additional variables potentially impacting children’s adjustment to school, such as teacher-child relationships, emotion regulation and language skills. Another limitation of this study is that information on school adjustment and peer relationships was obtained from a single source. The use of multiple sources of information (peers, parents, etc.) in future studies will further contribute to the validity of the findings. Lastly, in this study, children with typical development were examined, and their developmental assessments were conducted by their teachers. However, no developmental tests were administered by the researcher. This raises the possibility that children with undiagnosed developmental delays or autism spectrum disorder might have been included in the study. Future research should apply standard developmental tests to more accurately determine the developmental status of the children included, thereby ensuring more reliable and generalizable results.

Theory of mind skills are essential for children to develop positive social relationships, to develop skills in coping with social problem situations, and to adapt to school (Guven et al., 2019). Theory-of-mind-based educational intervention programs improve children’s theory of mind skills and prosocial behavior (Gozun Kahraman, 2012). In preschool classrooms, the frequent use of theory of mind concepts (belief, desire, intention, etc.) by teachers should be supported by enriched learning centers and activities (play, drama, storytelling, etc.) that allow children to use these concepts. It facilitates school adjustment by preventing negative peer relationships resulting from the lack and inadequacy of theory skills.

## Data availability statement

The raw data supporting the conclusions of this article will be made available by the authors, without undue reservation.

## Ethics statement

The studies involving humans were approved by the Kocaeli University Social and Humanities Ethics Committee, Türkiye (approval number: E-94094268-204.01.07-535197). The studies were conducted in accordance with the local legislation and institutional requirements. Written informed consent for participation in this study was provided by the participants’ legal guardians/next of kin.

## Author contributions

EU: Writing–original draft, Writing–review and editing.
